# Correction: Vaccination against the digestive enzyme Cathepsin B using a YS1646 *Salmonella enterica* Typhimurium vector provides almost complete protection against *Schistosoma mansoni* challenge in a mouse model

**DOI:** 10.1371/journal.pntd.0009936

**Published:** 2021-11-04

**Authors:** Adam S. Hassan, Nicholas H. Zelt, Dilhan J. Perera, Li Xing, Momar Ndao, Brian J. Ward

Li Xing should be included in the author byline. Li Xing should be listed as the fourth author and their affiliation is 2: the Research Institute of the McGill University Health Center (RI-MUHC), Montreal, Quebec, Canada. The contributions of this author are as follows: Conceptualization, Methodology, Project administration.

The correct citation is: Hassan AS, Zelt NH, Perera DJ, Xing L, Ndao M, Ward BJ (2019) Vaccination against the digestive enzyme Cathepsin B using a YS1646 *Salmonella enterica* Typhimurium vector provides almost complete protection against *Schistosoma mansoni* challenge in a mouse model. PLoS Negl Trop Dis 13(12): e0007490. https://doi.org/10.1371/journal.pntd.0007490
