# Supplementary figures and images for: Vaccination against the digestive enzyme Cathepsin B using a YS1646 Salmonella enterica Typhimurium vector provides almost complete protection against Schistosoma mansoni challenge in a mouse model
Source: PLoS Negl Trop Dis. 2019 Dec 2;13(12):e0007490. doi: 10.1371/journal.pntd.0007490 (PMC6907844; doi:10.1371/journal.pntd.0007490)

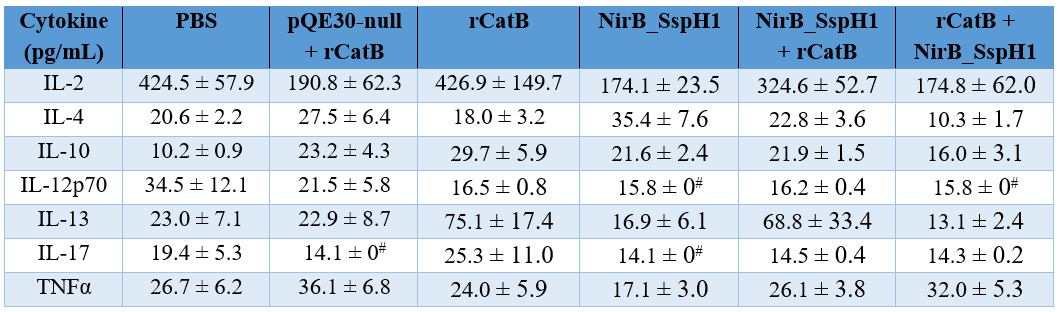

Supplement: S1 Table — Supernatant levels of different cytokines after stimulating splenocytes with rCatB for 72 hours were measured by QUANSYS multiplex ELISA. These results represent 5–7 animals per group. Results are expressed as the mean ± the standard error of the mean. #Values were below the limit of detection. (TIF) [file pntd.0007490.s001.tif]

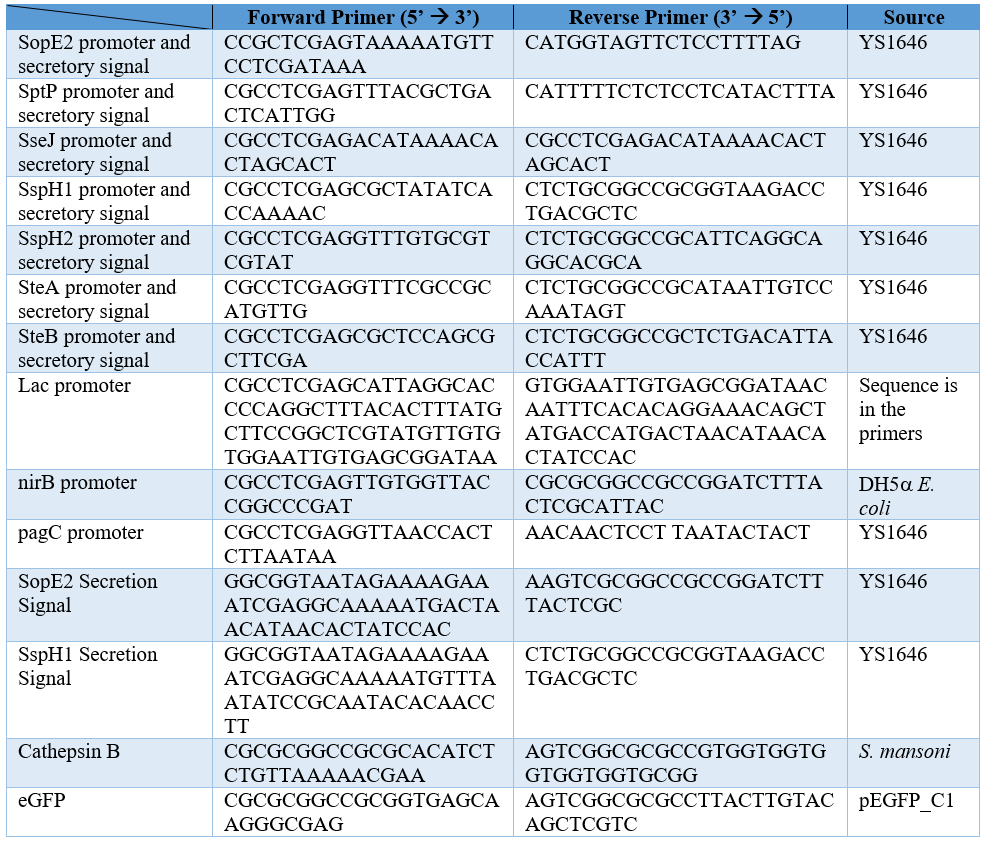

Supplement: S2 Table — (TIF) [file pntd.0007490.s002.tif]

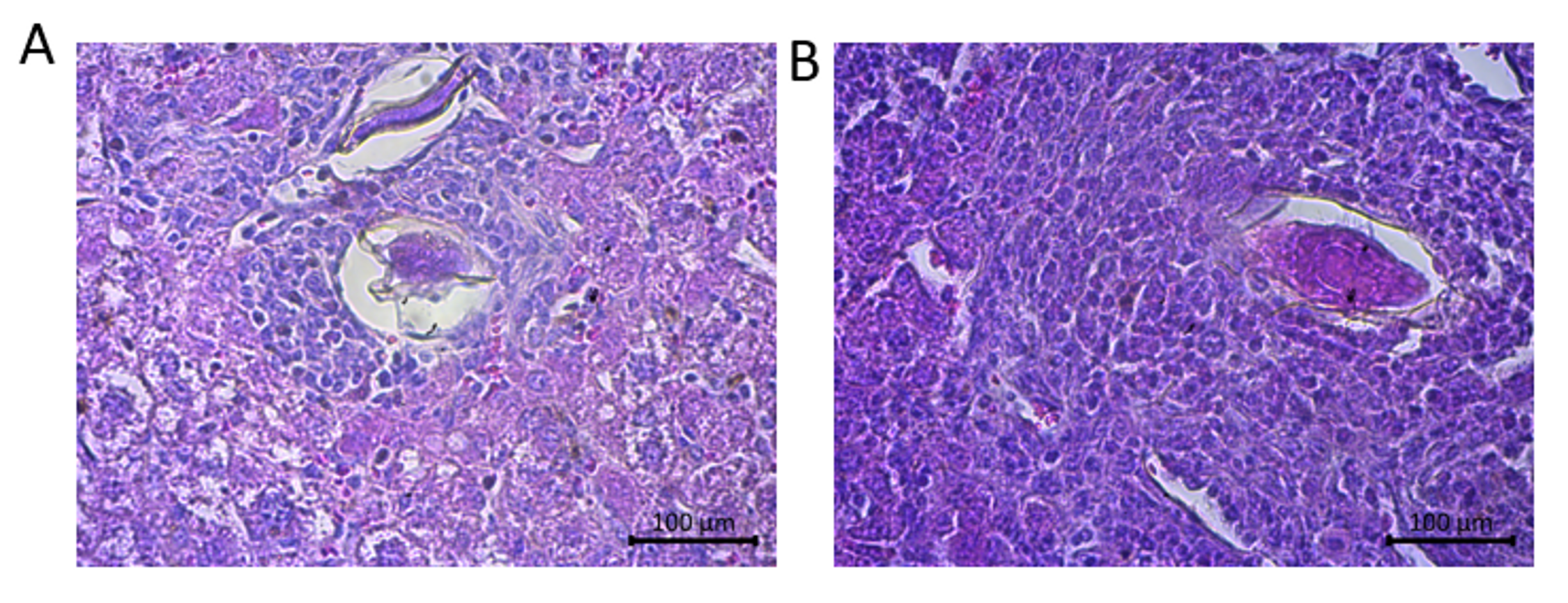

Supplement: S1 Fig — Representative images of H&E staining of granulomas from livers of vaccinated mice (A) and saline control mice (B). Panel A represents the PO → IM group for the nirB_SspH1 construct. Scale is set to 100 μm. (TIF) [file pntd.0007490.s003.tif]
